# 3-Hydroxyflavones and 3-Hydroxy-4-oxoquinolines as Carbon Monoxide-Releasing Molecules

**DOI:** 10.3390/molecules24071252

**Published:** 2019-03-30

**Authors:** Tatiana Soboleva, Lisa M. Berreau

**Affiliations:** Department of Chemistry & Biochemistry, Utah State University, 0300 Old Main Hill, Logan, UT 84322-0300, USA; tatiana.soboleva@aggiemail.usu.edu

**Keywords:** Gasotransmitter, mechanism, carbon monoxide, oxygen, heme oxygenase

## Abstract

Carbon monoxide-releasing molecules (CORMs) that enable the delivery of controlled amounts of CO are of strong current interest for applications in biological systems. In this review, we examine the various conditions under which CO is released from 3-hydroxyflavones and 3-hydroxy-4-oxoquinolines to advance the understanding of how these molecules, or derivatives thereof, may be developed as CORMs. Enzymatic pathways from quercetin dioxygenases and 3-hydroxy-4-oxoquinoline dioxygenases leading to CO release are examined, along with model systems for these enzymes. Base-catalyzed and non-redox-metal promoted CO release, as well as UV and visible light-driven CO release from 3-hydroxyflavones and 3-hydroxy-4-oxoquinolines, are summarized. The visible light-induced CO release reactivity of recently developed extended 3-hydroxyflavones and a 3-hydroxybenzo[*g*]quinolone, and their uses as intracellular CORMs, are discussed. Overall, this review provides insight into the chemical factors that affect the thermal and photochemical dioxygenase-type CO release reactions of these heterocyclic compounds.

## 1. Introduction

Advancing understanding of carbon monoxide (CO) as a signaling molecule in humans, and evaluating its potential application as a therapeutic agent, are significant current goals in biomedical research [1,2]. Whether endogenously generated or introduced as CO gas, low concentrations of CO produce vasodilation, anti-inflammatory and anti-apoptotic effects that suggest it could be useful in treating cardiovascular disease [3,4,5,6]. The delivery of controlled amounts of CO to tumor tissue is also suggested as an approach for treating cancer [7,8]. Providing a precise amount of CO gas to a specific tissue or organ is difficult. Thus, to enhance the spatiotemporal control of CO release, carbon-monoxide releasing molecules (CORMs) have been developed. The vast majority of CORMs reported to date that have been used in in vitro and in vivo studies are metal carbonyl compounds, with the [Ru(CO)_3_Cl_2_]_2_ dimer (CORM-2), and the water soluble molecules [Ru(CO)_3_Cl(glycinato)] (CORM-3) and [Mn(CO)_4_(S_2_CNCH_3_)(CH_2_CO_2_H)] (CORM-401), having been the most extensively employed [9,10]. Notably, the Ru(II) complexes exhibit complicated solution chemistry, including in blood plasma, and interact with proteins [11,12,13,14,15,16]. Thus, these compounds do not exhibit a single predictable formulation for CO delivery [16,17]. Complicating matters further is the fact that CO release from CORM-2, CORM-3 and CORM-401 occurs spontaneously via ligand exchange. This means that CO release begins when the compound is dissolved in a solution or buffer, and that there is no spatiotemporal control over CO delivery. Notably, recent studies also suggest that some biological effects that have been reported for the Ru(II)-containing CORMs and CORM-401 may not be due to CO release, but instead result from the reactivity of the metal complex or a fragment thereof [18,19]. These limitations provide the impetus for the development of new types of CO-releasing molecules that do not involve a metal carbonyl moiety. 

**Figure 1:**
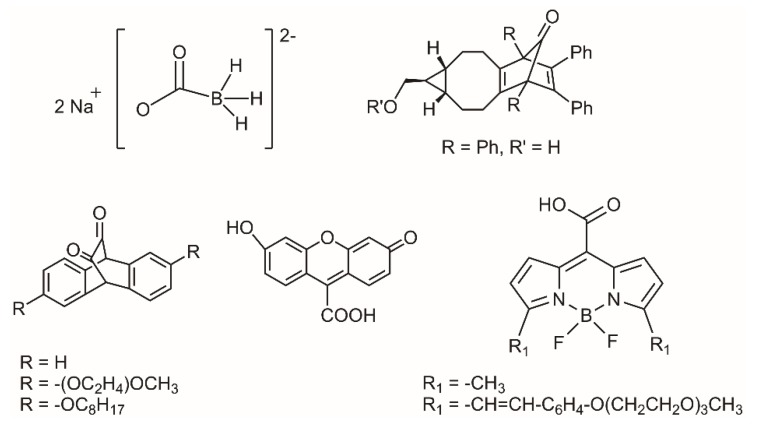
Examples of transition metal-free CO-releasing molecules (CORMs). Spontaneous (top) and visible light-induced (bottom) CORMs.

Several research labs have taken up the challenge of preparing transition metal-free CORMs [20]. Examples reported to date include sodium boronocarbonate (CORM-A1 and analogs) [21] and norbornadiene-7-one derivatives [22], both of which are spontaneous CO-releasing CORMs (Figure 1(top)). Visible light-induced organic photoCORMs reported to date include unsaturated cyclic diketones [23], xanthene carboxylic acid [24], and *meso*-carboxy BODIPY [25] (Figure 1(bottom)). Limitations of these transition metal-free CORMs and photoCORMs include low yield multi-step synthetic procedures and a lack of well-defined CO release products. Thus, despite these advancements, further work is needed. The development of a tunable structural framework for CO release, that enables modulation of key properties (e.g., solubility, trackability in cells, cellular or subcellular targeting, triggered CO release) via standard synthetic and medicinal chemistry approaches would advance the field. 

**Scheme 1:**
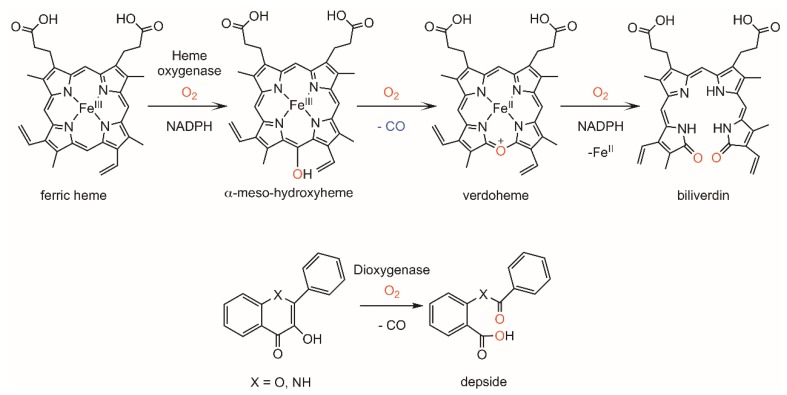
CO release reactions catalyzed by heme oxygenases (top) and 3-hydroxyflavone and 3-hydroxy-4-oxoquinoline dioxygenases (bottom).

To design a new family of CORMs, we considered how CO is generated in biological systems. In humans, CO is endogenously generated with an equimolar amount of biliverdin in the first step of O_2_-dependent heme degradation in a reaction catalyzed by heme oxygenases (HO-1 and HO-2, Scheme 1(top)) [26,27]. The expression of HO-1 protein is a response to cellular stress, with the products of heme degradation exhibiting notable biological effects. Specifically, CO modulates vascular tone and biliverdin/bilirubin function as potent antioxidants [28]. In fungi and bacteria, the O_2_-dependent cleavage of 3-hydroxyflavone and 3-hydroxy-4-oxoquinoline derivatives occurs via enzyme-catalyzed dioxygenase-type reactions in which a 2,4-peroxo species undergoes O-O and C-C bond cleavage to give one equivalent each of CO and a carboxylic acid byproduct termed a depside (Scheme 1(bottom)) [29,30]. The biological implications of CO production in fungi and bacteria could be for its use as carbon, energy, or electron source [31]. Overall the similar nature of the heme oxygenase and bacterial/fungal CO release reactions suggests that if controlled CO release reactivity from 3-hydroxyflavone and 3-hydroxy-4-oxoquinoline derivatives can be harnessed, compounds of this class will provide a novel family of CORMs. An attractive feature of this strategy is that flavonols are already under significant investigation for their beneficial health effects. For example, quercetin, a 3-hydroxyflavone derivative found in many fruits and vegetables, is noted for its potentially beneficial anti-inflammatory, antioxidant and anti-cancer properties [32,33,34]. Triggered 3-hydroxyflavone or 3-hydroxy-4-oxoquinoline-based CORMs that could be targeted to specific locations for CO release would be especially attractive for probing the localized effects of CO delivery [35]. Using such bioactive frameworks, it may be possible to deliver CO while producing additional beneficial effects. 

In this review we examine enzymatic and non-enzymatic reaction pathways leading to CO release from 3-hydroxyflavone and 3-hydroxy-4-oxoquinoline derivatives to elucidate conditions that influence CO release reactivity. Additionally, we discuss recent studies of photoinduced CO release from novel extended 3-hydroxyflavone and 3-hydroxybenzo[*g*]quinolone motifs. Overall, these combined results provide insight into the potential to control CO release from these heterocyclic frameworks and their potential for use as nature-inspired CORMs. 

## 2. CO Production from 3-Hydroxyflavones in Enzyme-Catalyzed Reactions and Model Systems

### 2.1. Fungal Flavonol Dioxygenases

**Scheme 2:**
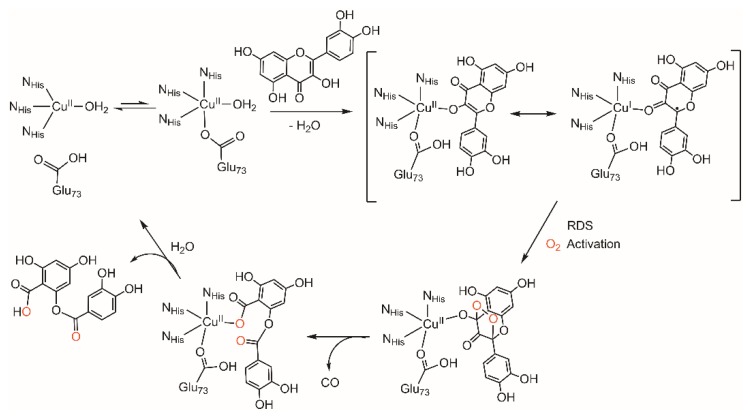
Proposed reaction pathway for CO release catalyzed by fungal flavonol dioxygenases [39]. RDS indicates the rate-determining step.

Soil microorganisms have divalent metal-containing enzymes that catalyze CO release from 3-hydroxyflavones. The flavonol dioxygenase from the fungus *A. japonicus* is a homodimer with a mononuclear Cu(II) center in the *N*-terminal domain [36]. Two different active site coordination geometries were identified for the Cu(II) center. Approximately 70% of the mononuclear Cu(II) sites exhibit a distorted tetrahedral coordination environment comprised of three histidine donors and a water molecule. The remaining Cu(II) centers (~30%) have an additional glutamate ligand (Glu_73_), resulting in an overall distorted trigonal bipyramidal geometry. EXAFS studies suggest that monodentate coordination of the substrate quercetin occurs with displacement of the water molecule [37]. As the enzyme exhibits 1000-fold lower activity in a site-directed mutant lacking Glu_73_, it has been suggested that this residue acts as an active site base for deprotonation of the flavonol 3-hydroxyl moiety [38]. The consensus reaction mechanism proposed for CO release involves the distorted square pyramidal five-coordinate Cu(II) enzyme/substrate complex forming a low-lying excited state Cu(I)-flavonoxy radical species that can undergo reaction with ^3^O_2_ either at the copper center or with the radical on the substrate (Scheme 2). Hybrid DFT calculations offer support for both pathways [39,40]. The number and location of hydroxyl substituents on the substrate determines the relative rate of enzymatic oxidation [41]. This relates both to the redox potential of the substrate and to secondary hydrogen bonding interactions that influence the positioning of the substrate in the active site [42]. 

### 2.2. Bacterial Flavonol Dioxygenases

Examples of flavonol dioxygenases from *Bacillus subtilis* [43,44,45,46] and *Streptomyces sp. FLA* [47,48,49,50,51,52] have been recently characterized. X-ray crystallographic studies of the iron-containing form of the *Bacillus subtilis* enzyme revealed a mononuclear five-coordinate Fe(II) center in each cupin domain of the bicupin protein [45]. Similar to the *A. japonicus* enzyme, the metal center is ligated by three histidine residues, a glutamate, and a water molecule. Initial kinetic studies of the *B. subtilis* enzyme as a function of metal ion provided an activity profile that has an approximate match to the Irving−Williams metal ion series for the stability of His- and Glu-ligated metal complexes [45]. Subsequent kinetic studies showed that inclusion of four different metal ions (Co(II), Mn(II), Cu(II), and Ni(II)) produced higher levels of turnover than the Fe(II)-containing enzyme, with the highest turnover in the presence of Mn(II) [46]. The *k*_cat_/*K*_M_ value and turnover number (~25 s^−1^) for the Mn(II)-containing *B. subtilis* enzyme is similar to that reported for the Cu^II^-containing *A. japonicus* enzyme. Both bind approximately two equivalents of metal ion, while the Co^II^ and Fe^II^-containing *B. subtilis* enzyme exhibit lower metal affinity as well as lower *k_cat_/K_M_* values. 

**Figure 2:**
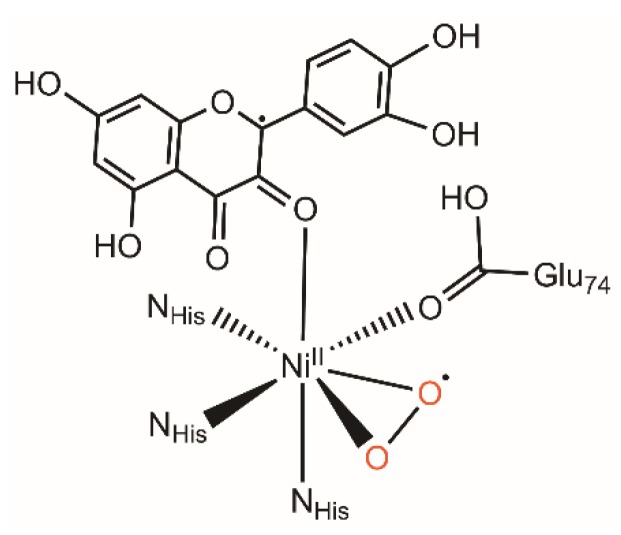
Representation of the active site features of cryotrapped *Streptomyces sp. FLA* in the presence of O_2_ [50].

**Scheme 3:**
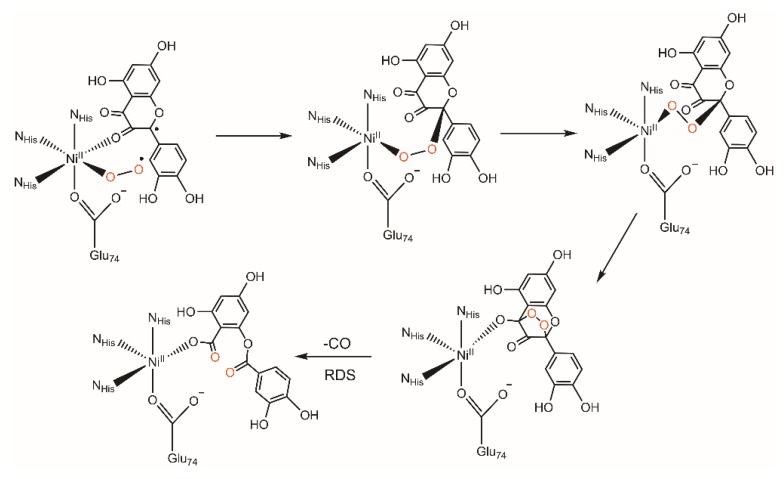
Proposed reaction pathway for CO release from Ni(II)-containing QDO from *Streptomyces sp. FLA* [52]. RDS indicates the rate-determining step.

The quercetinase from *Streptomyces sp. FLA* is a monocupin protein that exhibits the highest turnover in the presence of Ni(II) and Co(II) [48]. Notably, anaerobic absorption spectra of the Ni(II) or Co(II)-containing *Streptomyces sp. FLA* enzyme in the presence of the quercetin substrate showed a hypsochromic shifts for the flavonol lowest energy absorption feature (Band I). The opposite is observed for the Cu(II)-containing quercetinase of *Aspergillus flavus* wherein a bathochromic shift of the flavonol Band I is observed [49]. Considering redox potentials, the formation of transient Co(I)- or Ni(I)-flavonoxy radical species is unlikely in the reaction pathway of the *B. subtilis* and *Streptomyces sp. FLA* quercetin dixoygenases (QDOs). This led to the proposal of the active site metal ion having a non-redox role and instead acting as a conduit for electron transfer from the metal-bound flavonol substrate to coordinated dioxygen [46,48]. Evidence for an active site metal structure relevant to this proposal was found in the X-ray crystallographic analysis of a cryotrapped version of the Ni(II)-containing QDO from *Streptomyces sp. FLA* in the presence of O_2_ [50]. While exhibiting the typical coordination of three histidines and one glutamate to the Ni(II) center, a monodentate coordinated quercetin (via the 3-OH which is likely deprotonated at the pH = 8 optimum for this enzyme) and a side-on coordinated O_2_ are also present (Figure 2). These species are proposed to represent flavonoxy and superoxide radicals, respectively, having formed via electron transfer from the flavonolato anion to O_2_ via the Ni(II) conduit. The O_2_ ligand is perpendicular to the C2-C3 and C3-C4 bonds, which subsequently undergo oxidative cleavage leading to CO release from the C3-OH moiety. Quantum mechanics/molecular mechanics (QM/MM) simulations from two different laboratories have provided insight into the possible reaction mechanism for the Ni(II)-containing QDO [51,52]. Key differences between the two studies concern whether the active site glutamate (Glu_74_) is protonated and the assignment of the rate-determining step. With regard to the former, Li, et al. performed their investigations with a protonated Glu_74_ [51] whereas Wang, et al. investigated both protonation levels of Glu_74_ [52]. Both investigations suggest that dioxygen binding to the Ni(II) center results in electron transfer from the deprotonated flavonolato anion to O_2_ resulting in the formation of a Ni(II) flavonoxy radical/superoxide species with the O_2_^−^ ligand coordinated in an end-on fashion. The ground state of this complex is an open-shell singlet with a high-spin Ni(II) center antiferromagnetically coupled to the superoxide and flavonoxy radicals. In the singlet state, the O_2_ moiety coordinates in an end-on fashion which differs from the observed side-on binding in the X-ray structure [50]. Attack of the terminal superoxide oxygen atom at C2 of the flavonoxy radical leads to the formation of a bridging Ni(II)-peroxo species. Li, et al. suggest that a conformational change involving movement of the proximal Ni(II)-coordinated peroxo oxygen atom closer to the C4 atom of the flavonolato ligand is the rate-determining step, with a free energy barrier of 19.9 kcal/mol [51]. However, the calculations of Wang, et al. instead suggest that this step is not rate-determining and proceeds to two different intermediates that differ in terms of the orientation of the glutamate residue. In both calculations, subsequent formation of a five-membered cyclic intermediate via C4-O bond formation enables concurrent C2-C3 and C3-C4 bond cleavage, extrusion of CO, and formation of the depside product (Scheme 3). Wang, et al. propose that cleavage of the cyclic peroxide is rate-determining, with a free energy barrier of 17.4 kcal/mol, which is similar to experimental kinetic data (~15 kcal/mol) [48]. The calculations of Wang, et al. also indicate that if the glutamate residue is protonated, cleavage of the C2-C3 and O-O bonds should occur leading to the formation of a α-keto acid product, which has not been experimentally observed [52]. 

**Scheme 4:**
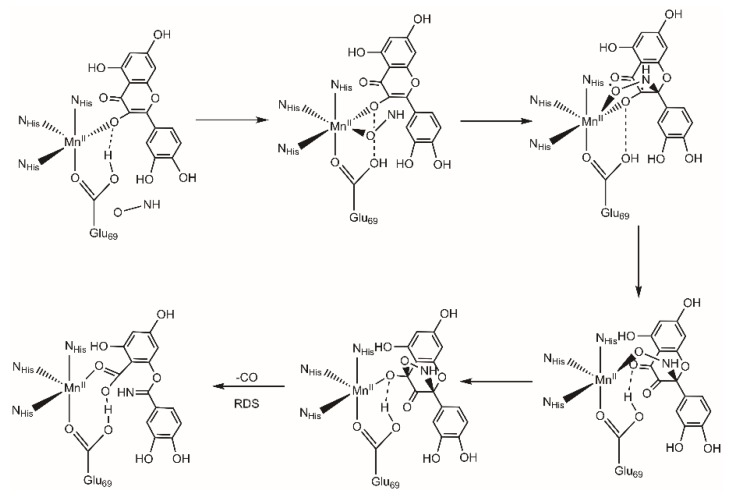
Mechanism proposed for Mn(II)-containing bacterial QDO reactivity with HNO based on quantum chemical studies [55].

As outlined above, bacterial quercetin dioxygenases (QDOs) catalyze the O_2_-dependent cleavage of flavonols to release CO and the corresponding depside. Recently, Farmer and co-workers have shown that the Mn-containing QDO from *Bacillus subtilis* will catalyze CO release from quercetin in the presence of a different small molecule, nitrosyl hydride (HNO) [53]. This nitroxygenase activity results in the incorporation of N and O atoms in the depside product (Scheme 4). The substitution of dioxygen with nitrosyl hydride is rationalized on the basis of the fact that the anionic form of nitrosyl exists as a triplet (^3^NO^−^) and is isoelectronic with dioxygen [54]. The Mn-QDO nitroxygenase reactivity was found to be highly regioselective, producing only 2-((3,4-dihydroxyphenyl)(imino)methoxy)-4,6-dihydroxybenzoate (Scheme 4). Although dioxygenase activity is found for the Fe(II)- or Co(II)-containing QDO enzymes, no nitroxygenase activity was found for these enzymes [53]. The nitroxygenase reaction pathway has been evaluated quantum chemically by Wojdyla and Borowski [55]. The lowest energy reaction sequence involves initial Mn(II)-O-NH complex formation. Addition of the NH moiety of the Mn(II)-nitroxyl to the flavonolato C2 center, followed by shifting of the hydrogen bond involving Glu_69_ from O3 to O4, enables C4-O bond formation (Scheme 4). The rate-determining step, with a barrier of 21.5 kcal/mol, involves cleavage of the five-membered ring leading to CO extrusion. The regioselectivity of the reaction is an inherent property of the reactants. Specifically, the larger electrophilicity of the nitrogen atom in HNO makes the C2-N bond formation more feasible than C2-O, with the C2-N quercetin:HNO adduct being >15 kcal/mol lower in energy versus the C2-O adduct. 

### 2.3. Model Systems for Cu(II)-Containing Fungal QDOs

**Scheme 5:**
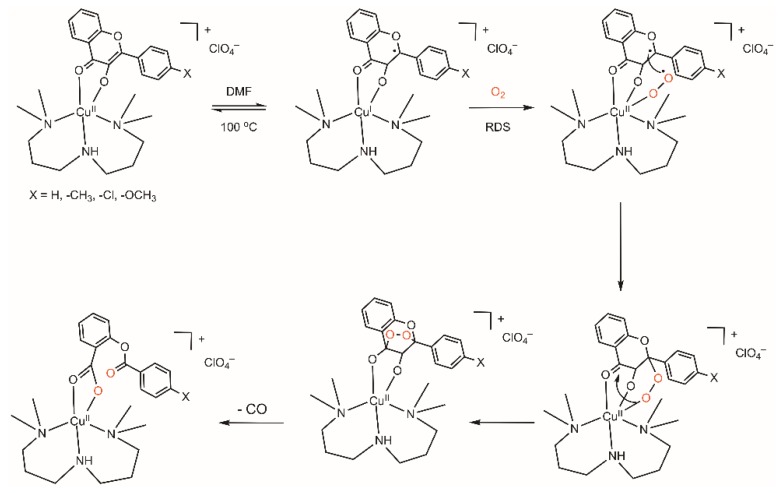
Reactivity of [Cu(idpa)(fla)]ClO_4_ derivatives with O_2_ to release CO [59].

Many synthetic model complexes have been reported for the enzyme/substrate adduct in fungal copper-containing quercetin dioxygenases, with many notable contributions from Speier and co-workers [56,57,58]. These Cu(II) complexes typically contain a supporting bidentate or tridentate nitrogen donor ligand to mimic histidine ligation and a bidentate-coordinated flavonolato ligand involving a five-membered chelate ring formed via coordination of the deprotonated 3-OH moiety and the 4-keto oxygen atom. This flavonolato coordination mode is different from the monodentate coordination proposed in the enzyme/substrate complex of the *A. japonicus* enzyme, which is suggested to result from secondary interactions involving the quercetin substrate hydroxyl substituents [36,37]. The synthetic Cu(II) flavonolato complexes are much less reactive than the enzyme, typically undergoing O_2_-dependent dioxygenase-type reactivity only at elevated temperatures (e.g., 80–120 °C) in DMF. Enzyme-like products are generated, specifically *O*-benzoylsalicylate (depside) which is often retained on the Cu(II) center (Scheme 5), and CO, which is typically not quantified. A bimolecular rate law for such reactions is –d[Cu(II) flavonolato complex]/dt = *k*[Cu(II) flavonolato complex][O_2_]. An example of such a reaction is the O_2_-dependent CO release reaction of [Cu(idpa)(fla)]ClO_4_ (Scheme 5; fla = monoanion of 3-hydroxyflavone; idpa = 3,3’-imino-bis(*N*,*N*-dimethylpropylamine)) [59]. Similar to the proposed enzyme reaction pathway, an idpa-coordinated Cu(I)-flavonoxy radical species is suggested to undergo single electron transfer from Cu(I) to O_2_ to form a Cu(II) superoxide adduct. The terminal oxygen atom of superoxide moiety subsequently combines with the flavonoxy radical to form a bridging peroxo species. Attack of the Cu(II)-coordinated peroxo oxygen atom at C4 results in the formation of a 2,4-cyclic peroxide species from which CO extrusion and depside formation can occur. For this reaction, Δ*H*^‡^ = 64 ± 5 kJ/mol and Δ*S*^‡^ = −120 J/mol·K. The negative entropy of activation is consistent with the O_2_ activation step to produce a Cu(II)-superoxo species being rate-determining. Enhanced electron density within the flavonolato ligand increases the oxygenation rate with the Hammett ρ value being −0.29. 

**Scheme 6:**
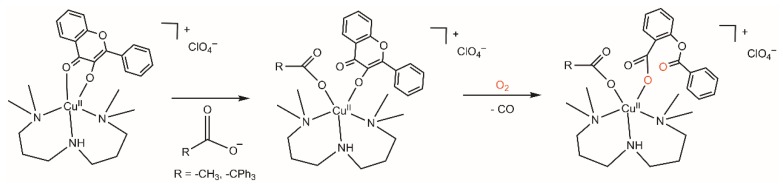
Effect of carboxylate addition on flavonolato coordination [60].

Notably, addition of an exogenous carboxylate donor ligand (acetate or triphenylacetate) to solutions of [Cu(idpa)(fla)]ClO_4_ results in an acceleration of the CO release reaction [57,60]. This is attributed to Cu(II) coordination of the carboxylate ligand, which results in a shift to monodentate coordination of the flavonolato ligand (Scheme 6). The enhanced electron density within the flavonolato moiety at C2 makes direct electron transfer to O_2_ more viable thus enhancing the rate of formation of O_2_^-^, which was detected in reaction mixtures. 

**Scheme 7:**
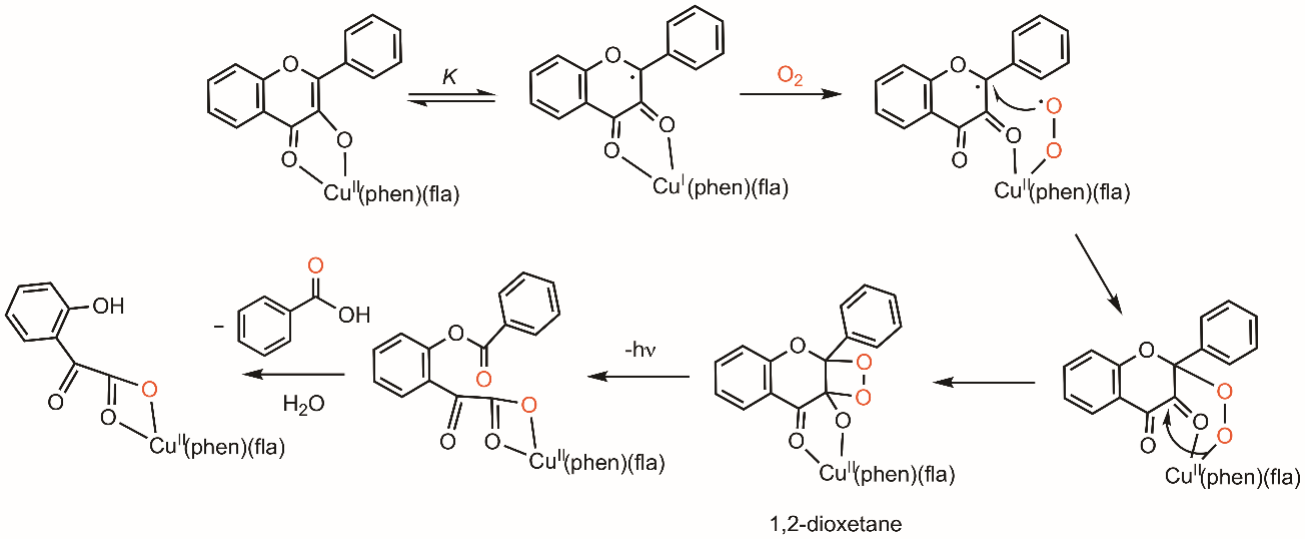
O_2_ reactivity of a phenanthroline-coordinated Cu(II) flavonolato complex that does not result in CO release [61].

Some Cu(II) flavonolato complexes, such as [Cu(phen)(fla)_2_] and [Cu(bpy)(fla)_2_], do not exhibit CO release but instead undergo reaction via a 1,2-dioxetane intermediate to give 2-hydroxyphenylglyoxylate following hydrolysis (Scheme 7) [61,62]. The ligand environment and Lewis acidity of the Cu(II) center are suggested to affect the regioselectivity of carbon-carbon bond cleavage by influencing the electrophilicity of the flavonolato carbonyl center [61]. Evidence for the involvement of a 1,2-dioxetane are emission bands at 506, 546, and 578 nm [62]. 

### 2.4. Model Systems for Bacterial QDOs

**Scheme 8:**
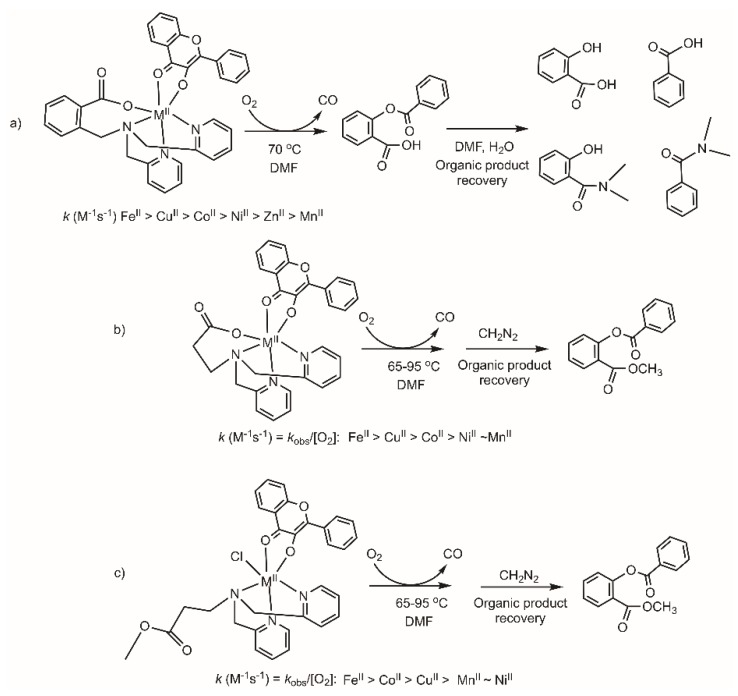
Dioxygenase-type reactivity of divalent metal complexes containing a carboxylate or ester appendage [64,65].

Grubel, et al. synthesized the first series of divalent 3*d* metal flavonolato compexes of structural relevance to bacterial quercetin dioxygenases [63]. These complexes, supported by an aryl-appended tris(pyridylmethyl)amine ligand, have thus far not been investigated for thermal CO release reactivity. Sun, et al. and Matuz, et al. subsequently prepared, characterized, and examined the O_2_-dependent CO release reactivity of two series of divalent metal ion flavonolato complexes (Scheme 8) [64,65]. Sun, et al. studied complexes supported by a carboxylate-containing N_3_O-donor ligand (Scheme 8a) [64]. A metal ion dependence on the second-order rate constant for oxidative cleavage (Fe(II) > Cu(II) > Co(II) > Ni(II) >Zn(II) > Mn(II)) correlates with the oxidation potential of the coordinated flavonolato ligand, which in turn is influenced by the Lewis acidity of the metal center. These oxidative cleavage reactions occurred at lower temperatures than those reported for synthetic complexes having a neutral nitrogen supporting ligand environment [65], likely due to the reduced Lewis acidity of the metal center. The yield of CO produced using the Fe(II) complexes supported by carboxylate-containing N_3_O-donor ligand was determined to be 68%. The series of complexes reported by Matuz, et al. (Scheme 8b,c exhibit similar reactivity in terms of the products generated [65]. The ester-appended derivatives shown in Scheme 8c are more reactive that the propionate analogs (Scheme 8b), suggesting that ligand exchange involving the monodentate anion is important in the rate-determining O_2_ activation step. 

**Scheme 9:**
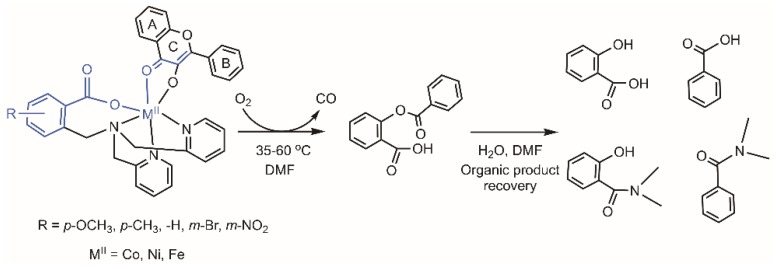
Electronic effects on flavonolato ligand reactivity with O_2_. The blue highlighted portion of the structure shows the benzoate-M(II)-O(4)=C(27)-C(21)=C(22) conduit [66,67,68].

Enhancing the electron density within the carboxylate moiety of the supporting chelate ligand produces structural, electronic and reactivity effects via the benzoate-M(II)-O(4)=C(27)-C(21)=C(22) conduit in M(II)-containing flavonolato complexes. Specifically, incorporation of an electron donating group on the ligand benzoate moiety produces a smaller torsion angle between the flavonol B and C rings (Scheme 9), a π→π* absorption band that is shifted to lower energy, and a lower redox potential for the flavonolato moiety. These characteristics result in higher dioxygenase reactivity in Co(II), Ni(II) and Fe(II) complexes [66,67,68]. CO quantification studies were not reported for these reactions. 

### 2.5. CO-Release Reactivity of Other Metal Flavonolato Complexes

**Figure 3:**
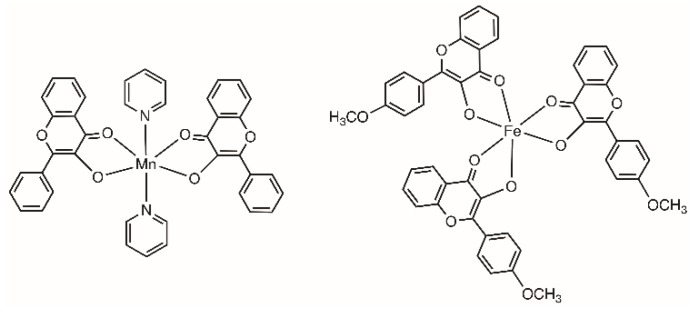
Mononuclear Mn(II) and Fe(III) flavonolato complexes that undergo CO release in DMF [69].

Simple metal flavonolato complexes that lack structural relevance to quercetin dioxygenase but exhibit CO release reactivity have also been reported by Speier and co-workers [56,69,70,71]. When heated in DMF at 95 °C, bis- and tris-flavonolato complexes such as Mn(fla)_2_(py)_2_ and Fe(4′-MeO-fla)_3_ (Figure 3) release CO in near quantitative yields (80–90%) resulting in the formation of the corresponding *O*-benzosalicylate complexes. The rate law for these reactions is –d[complex]/d*t* = *k*[complex][O_2_], with the rate constant for the Mn(II) derivative being approximately 6-fold larger than that of the Fe(II) complex (0.50 and 0.08 M^−1^·s^−1^, respectively) at 100 °C in DMF. 

**Scheme 10:**
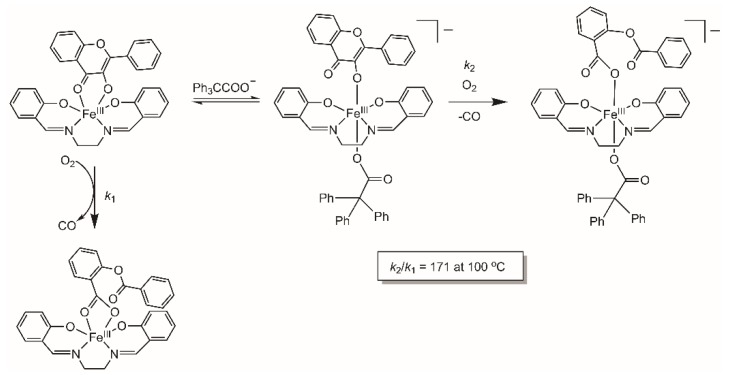
Effect of bulky carboxylate ligation on the dioxygenase-type CO release reactivity of an Fe(III) flavonolato complex [72].

Anion effects on the O_2_ reactivity of Fe(III) flavonolato complexes have been reported [72]. The Fe(III) flavonolato complex Fe(fla)(salen) (fla = anion of 3-hydroxyflavone, Scheme 10) undergoes reaction with O_2_ at 100–125 °C in DMF to give an *O*-benzosalicylate complex and CO [72]. The rate law for this reaction is –d[Fe(fla)(salen)]/d*t* = *k*[Fe(fla)(salen)][O_2_] with *k* = 2.07 ± 0.12 M^−1^·s^−1^, Δ*H*^‡^ = 76 kJ/mol and Δ*S*^‡^ = −94 J/mol·K at 373.16 K. Incorporation of an electron-donating group at the *para* position of the flavonol results in a ~2.5-fold increase in the rate of reaction. A significant 171-fold rate enhancement is produced upon addition of a bulky carboxylate anion (triphenyl acetate, Ph_3_CCOO^−^, Scheme 10). The coordination of this anion to the Fe(III) center is proposed to induce a shift in the flavonolato coordination mode from bidentate to monodentate. The enhanced electron density within the flavonolato ligand is suggested to be responsible for the dramatic rate enhancement. The overall rate law for the anion-containing reaction is Rate = *k*[Fe(fla)(salen)][O_2_][Ph_3_CCOO^−^] with Δ*H*^‡^ = 35 kJ/mol and Δ*S*^‡^ = −120 J/mol·K at 313.16 K. This lower activation barrier results in a dioxygenase-type CO release reaction that can proceed at room temperature. 

### 2.6. Summary

Studies of quercetin dioxygenases and model systems for these enzymes have provided insight into the factors that influence the reactivity of metal-flavonolato species with O_2_ in dioxygenase-type CO-releasing reactions. In model complexes containing a flavonolato ligand coordinated in a bidentate manner via a five-membered chelate ring, high temperatures are typically required to induce O_2_-dependent CO release reactivity. Key to increasing this reactivity is to enhance the electron density within the flavonolato moiety, as this lowers the activation barrier for electron transfer for O_2_. Enhanced electron density can be achieved by adding electron-donating substituents on the flavonolato ligand, increasing the electronic donor properties of a supporting chelate ligand, or by introducing additional bulky anionic donors that via coordination to the metal center induce a shift to monodentate metal-flavonolato coordination. To date, the systems that exhibit reactivity closest to room temperature involve monodentate flavonolato coordination similar to that proposed in the enzymatic reaction pathways. In organic solvents, few of these complexes have been evaluated in terms of quantification of the amount of CO released. Additionally, 3*d* metal flavonolato complexes have not been evaluated as CO-releasing molecules in aqueous environments. Under such conditions, ligand exchange with water would likely influence the CO release reactivity. 

## 3. CO Production from 3-Hydroxy-4-oxoquinolines via Cofactor-Free Enzyme-Catalyzed Reactions and in Metal-Containing Synthetic Systems

### 3.1. Enzyme-Catalyzed Reactions

**Scheme 11:**
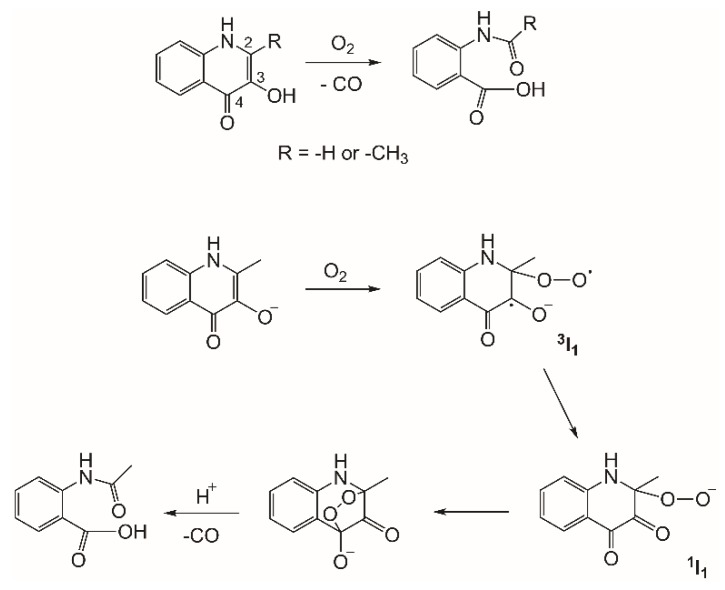
(top) Reaction catalyzed by 1*H*-3-hydroxy-4-oxoquinaldine 2,4-dioxygenase (R = -CH_3_; Hod) and 1*H*-3-hydroxy-4-oxoquinoline 2,4-dioxygenase (R = -H; Qdo). (bottom) Proposed reaction pathway of Hod based on kinetic, computational and spectroscopic studies.

The cofactor-free bacterial 1*H*-3-hydroxy-4-oxoquinaldine 2,4-dioxygenase (Hod) from *Arthrobacter ilicis* Rü61a and 1*H*-3-hydroxy-4-oxoquinoline 2,4-dioxygenase (Qdo) from *Pseudomonas putida* 33/1 catalyze the insertion of dioxygen at C2 and C4 of the 3-hydroxy-4-oxoquinoline substrates resulting in the release in CO (Scheme 11(top)) [73,74,75,76,77]. In terms of sequence, these enzymes are rare examples of dioxygenases that belong to the α/β hydrolase family. Hod and Qdo do not contain a cofactor or metal ion but have a catalytically required histidine residue (His_251_ in Hod; His_244_ in Qdo). The enzyme-bound monoanion of 1*H*-3-hydroxy-4-oxoquinaldine is proposed to undergo single electron transfer to O_2_ in the rate-determining step to produce a triplet superoxide complex (^3^I_1_, Scheme 11(bottom)). An internal electron transfer leads to a closed-shell singlet (^1^I_1_). Attack of the peroxide terminal oxygen at C4 followed by formation of a peroxide bridge between C2 and C4 leads to the cyclic species from which CO extrusion can occur to give *N*-acetylanthranilate anion. This reaction pathway is consistent with ^18^O_2_ labeling studies, which showed that the catalytic reaction of Hod results in incorporation of two labeled oxygen atoms. It should be noted that the substrates for Hod and Qdo exhibit similar reactivity under basic, aerobic conditions (vide infra). The role of the enzyme in these systems may be to provide a low-dielectric reaction environment that is optimal for positioning the substrate and for electron transfer to O_2_ and C-O bond formation. 

### 3.2. Synthetic Systems

**Scheme 12:**
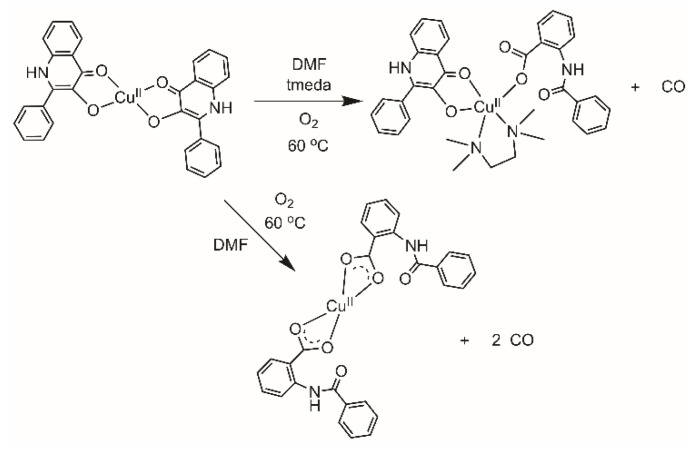
Reactions of a Cu(II) bis 1*H*-2-phenyl-3-hydroxy-4-oxoquinolinate complex with O_2_ to release CO [78].

While not structurally relevant to the active site chemistry of Hod, copper complexes containing a 1*H*-2-phenyl-3-hydroxy-4-oxoquinolinate ligand have been shown to undergo O_2_-dependent CO release [78]. As shown in Scheme 12, similar to the reactivity of Cu(II) flavonolato complexes, these complexes undergo stoichiometric CO release from each 3-hydroxy-4-oxoquinoline ligand upon heating in DMF. 

**Scheme 13:**
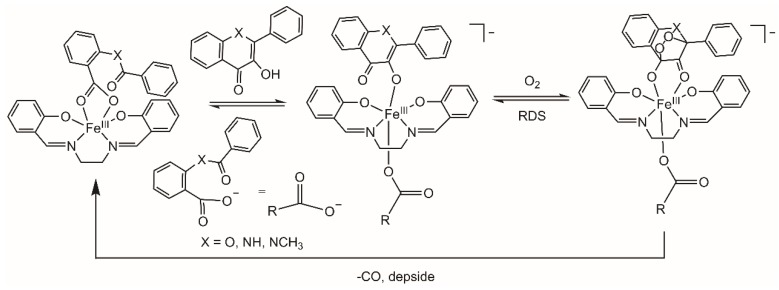
Oxygenation of 3-hydroxyflavone and 3-hydroxy-4-oxoquinoline derivatives with the release of CO catalyzed by [Fe(III)(*O*-bs)(salen)] [79].

A non-heme iron catalyst has been reported by Speier, et al. to mediate the O_2_-dependent cleavage of 3-hydroxyflavone and 1-*H*-2-phenyl-3-hydroxy-4-oxoquinoline to produce CO [79]. At 100 °C in DMF and with a ratio of 1:25 between the complex, [Fe(III)(O-bs)(salen)] (Scheme 13), and the substrate, catalytic dioxygenation occurs to give the products, *O*-benzoylsalicylic acid and *N*-benzoylanthranilic acid in 78% and 64% yield, respectively, with concomitant release of CO. Initial rate studies showed that 3-hydroxyflavone is three-times more reactive than 2-phenyl-3-hydroxy-4(1*H*)-quinoline toward O_2_. As expected, increasing the basicity of the flavonol increases the rate of the O_2_ reaction. These reactions proceed by single electron transfer (SET) wherein the metal-coordinated deprotonated substrate provides an electron for the reduction of O_2_. Superoxide formation was detected using nitroblue tetrazolium (NBT) as a scavenger to produce formazan. Proceeding through the reaction pathway as shown in Scheme 13, an endoperoxide intermediate is formed that undergoes C-C and O-O bond cleavage to release CO and generate an enzyme-type organic byproduct. The formation of *O*-benzoylsalicylate or *N*-anthranilate as products enhances the rate of reaction through coordination to the Fe(III) center as previously described. 

### 3.3. Summary

Overall these combined studies indicate that 3-hydroxy-4-oxoquinoline derivatives undergo enzyme-catalyzed or metal-promoted reactions to release *N*-benzoylanthranilic acid and an equivalent of CO. This clean reactivity suggests that 3-hydroxy-4-oxoquinolines may also be useful as a CO-releasing motif. Notably, the slower rate of CO release of 2-phenyl-3-hydroxy-4(1*H*)-quinoline derivatives versus 3-hydroxyflavones upon reaction with O_2_ indicates that the heteroatom within the pyrone ring is a key factor in determining the rate of CO release. 

## 4. CO Production from 3-Hydroxyflavones and 3-Hydroxy-4-oxoquinolines via Base-Catalyzed and Non-Redox Metal Assisted Reactions

### 4.1. 3-Hydroxyflavones

**Scheme 14:**
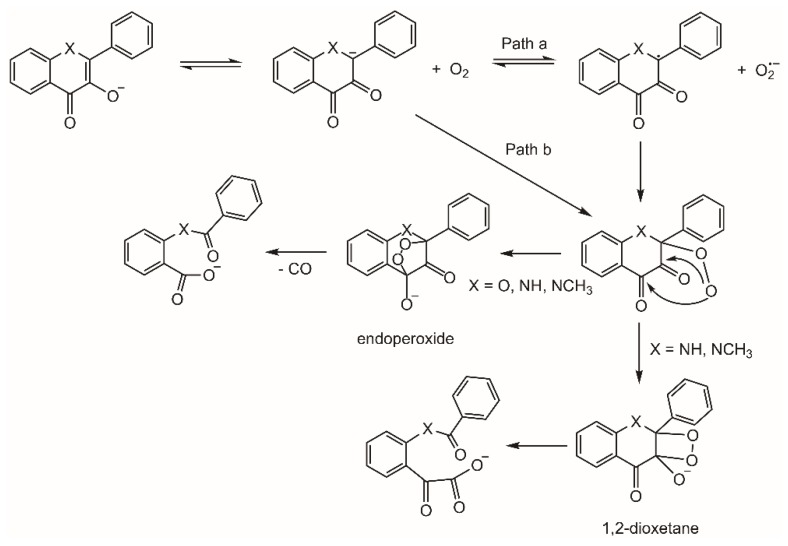
Reaction pathways leading to CO release in base-catalyzed and non-redox metal-promoted dioxygenation reactions of 3-hydroxyflavones and 3-hydroxy-4-oxoquinolines.

Speier, et al. have previously summarized base-catalyzed flavonol oxygenation that results in CO release in protic solvents as well as non-redox metal-assisted CO release reactions in a variety of solvents [56,80,81,82]. Briefly, flavonol oxygenation under these conditions (40–90 °C) proceeds via one of two mechanistic pathways: single electron transfer (SET) (Scheme 14(Path a)) or a one-step electrophilic reaction of triplet oxygen with the flavonolate ion (Scheme 14(Path b)). The former was found to occur in aprotic solvents via SET from the flavonolate ion to triplet O_2_, yielding a flavonoxy radical and superoxide ion, which subsequently undergo fast radical-radical coupling to give a 2-hydroxyperoxyflavan-3,4-dionate intermediate. The resulting species then undergoes an intramolecular nucleophilic attack at C4 leading to the formation of an unstable endoperoxide that decomposes into depside (*O*-benzoylsalicylate) and CO [81]. In the case of the electrophilic reaction between oxygen and the flavonolato ion, a detailed mechanistic study was carried out with 4’-substituted flavonol derivatives in 50% DMSO-H_2_O in the pH range 6.4–10.8 to map out the reaction pathway [82]. This reaction showed specific base catalysis. A linear Hammett plot yielded a negative reaction constant (ρ = −0.50) implying that a higher electron density on the flavonolate ion makes it more nucleophilic thus facilitating electrophilic attack by O_2_. Both oxygenation pathways yield stoichiometric formation of CO and depside and/or its hydrolysis byproducts (salicylic acid and benzoic acid). 

Farmer, et al. have reported the base-catalyzed reaction of HNO with quercetin [83]. Above pH 7, this reaction occurs to give 3,4-dihydroxybenzonitrile, a cleavage product indicating that the regioselectivity observed in the reaction catalyzed by the Mn-containing QDO from *Bacillus subtilis* (Scheme 4) is maintained in the base-catalyzed reaction. Kinetic and thermodynamic studies by Kumar and Farmer have provided evidence that the base-catalyzed reaction between HNO and quercetin occurs via single electron transfer (SET) [83]. It is notable that this reaction occurs orders of magnitude faster than the corresponding dioxygenation. This has been attributed to the difference in driving force for SET in the rate-determining step. 

### 4.2. 3-Hydroxy-4-oxoquinolines

For 3-hydroxy-4-oxoquinoline derivatives, a mixture of products resulting from endoperoxide or 1,2-dioxetane species forms, with the ratio depending on the solvent. CO release only occurs via the reaction involving the endoperoxide, with the 1,2-dioxetane undergoing C-C bond cleavage to give a phenylglyoxalic acid derivative (Scheme 14). A long-lived radical is observed in the reaction of 1-*H*-2-phenyl-3-hydroxy-4-oxoquinoline with O_2_ providing evidence for a single electron transfer pathway in the oxygen activation step [84]. 

### 4.3. Summary

Overall, the 3-hydroxyflavone derivatives exhibit cleaner reactivity under base-catalyzed conditions, whereas 3-hydroxy-4-oxoquinoline derivatives exhibit competing formation of 1,2-dioxetane species. This suggests that 3-hydroxyflavones may be more reliable CO release agents under biological conditions. 

## 5. CO Production from 3-Hydroxyflavones via Photochemical Reactions

**Scheme 15:**
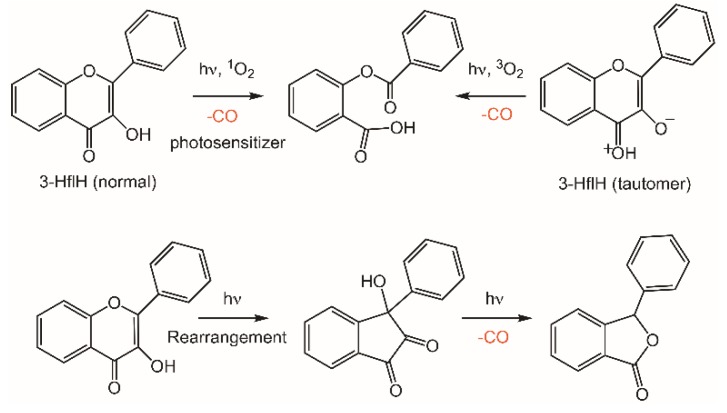
UV-light induced reactivity of 3-hydroxyflavone in the presence (top) and absence (bottom) of oxygen.

Photooxygenation of flavonols is another reaction pathway that results in CO release. It is known that unsubstituted 3-hydroxyflavone (3-HflH) will undergo incorporation of both oxygen atoms of O_2_ and expulsion of CO in the presence of a photosensitizer, or via direct illumination using UV light (Scheme 15 (top)) [85,86]. The latter is suggested to proceed via the reaction of a tautomeric triplet state (formed via excited state intramolecular proton transfer, ESIPT) with ground state ^3^O_2_ to give a 5-membered cyclic endoperoxide intermediate from which CO extrusion occurs [87,88,89]. A similar reaction pathway has been recently proposed for the photooxygenation of 4′-diethylamino-3-hydroxyflavone [90]. Notably, 3-hydroxyflavone is also known to undergo photoinduced rearrangement in the absence of O_2_ (Scheme 15 (bottom)) in apolar, polar aprotic and polar protic solvents, to give a 3-hydroxy-3-aryl-indane-1,2-dione product [91,92,93,94]. In some cases, the indane-1,2-diones releases CO to form a 3-arylphthalide (Scheme 15).

**Scheme 16:**
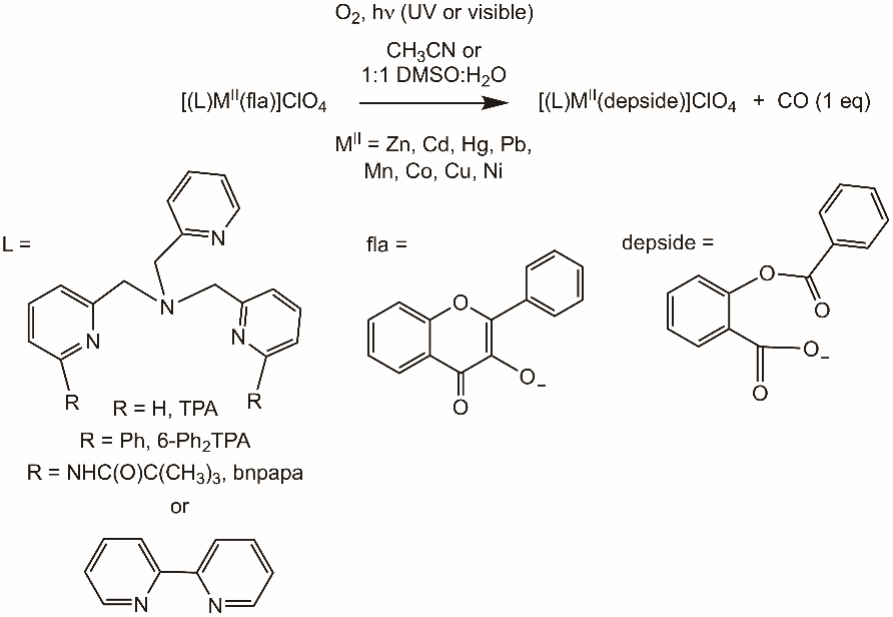
UV- or visible-light-induced CO release reactivity of divalent metal flavonolato complexes.

Our laboratory has recently demonstrated that divalent metal complexes of the general formula [(L)Zn(3-Hfl)]ClO_4_, containing a coordinated 3-hydroxyflavonolato ligand and supported by a chelating nitrogen donor ligand (L), undergo clean UV- or visible light-induced reactivity in the presence of O_2_ to produce one equivalent of CO (determined by GC head space analysis) and a metal carboxylate (depside) complex (Scheme 16) [95,96,97,98,99]. The reaction quantum yield (Table 1) for CO release from these complexes depends on the ligand secondary environment of the metal complex or solvent conditions, and on the divalent metal ion present. Divalent zinc 3-hydroxyflavonolato complexes of a variety of supporting chelate ligands exhibit reaction quantum yields for CO release that can be tuned from ~0.0003 to 0.012 [95,98,99]. Encapsulation of the Zn(II)-flavonolato moiety within a hydrophobic microenvironment produced the highest reaction quantum yield whereas positioning the Zn(II) 3-hydroxyflavonolato unit within a hydrogen bond donor pocket produced the least reactive complex. The latter may be explained in that hydrogen-bonding involving either rigid donors in the secondary environment, or a hydrogen bond donor solvent (e.g., H_2_O), provides additional pathways for non-radiative decay thus leading to excited state quenching and a less efficient CO release [98]. Su, et al. recently reported a series of zinc flavonolato complexes supported by tetradentate tripodal nitrogen donor ligands that are similar to those reported by our laboratory and release one equivalent of CO upon visible light illumination [100]. We note that Protti, et al. previously reported that the presence of O_2_ enhanced the photodecomposition of simple [Zn(3-Hfl)]^+^ species but did not report CO production [101]. 

The coordination of the 3-hydroxyflavonolato ligand to heavy closed shell metal ions (Cd^II^ < Hg^II^, and Pb^II^) produced relatively high quantum yields (>0.20) for CO release [95,96]. Conversely, coordination of the 3-hydroxyflavonolato anion to Mn(II), Co(II), Ni(II), or Cu(II) yielded complexes with low CO release reaction quantum yields (typically ~0.005) due to quenching of the excited state by the open-shell first row metal ion [96]. Despite the low reactivity, these complexes can serve as catalysts for the UV light-induced oxidative degradation of 3-hydroxyflavone to give *O*-benzoylsalicylic acid and CO [96]. While multiple catalysts for the thermal O_2_-dependent degradation of 3-hydroxyflavone with CO release have been reported [79,102,103], to our knowledge this is the only light-driven catalytic reaction reported to date. We also note that a Ag(I) 3-hydroxyflavonolato complex, [Ag(3-Hfl)(PPh_3_)_2_], was recently reported to undergo visible light-induced oxygenation to yield a depside complex [104]. No reaction quantum yield or CO quantification was reported for this reaction.

**Scheme 17:**
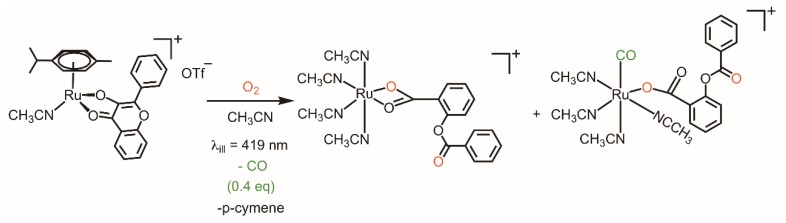
Reactivity of Ru^II^ 3-hydroxyflavonolato complex with O_2_ upon illuination with visible light (λ_ill_ = 419 nm) [105].

We have also explored the visible light-induced CO release reactivity of the [Ru(η^6^-*p*-cymene)(CH_3_CN)(3-Hfl)]OTf complex (Scheme 17) [105], as such complexes are promising for anticancer applications [106,107,108]. The combined results from product identification (ESI-MS), CO quantification, and ^18^O-isotope labeling studies upon illumination with UV (300 nm) and visible (419 nm) light in CH_3_CN show that oxygenation of the flavonolato ligand occurs in a dioxygenase-type manner with release of CO. However, the amount of free CO generated was found to depend on the wavelength of illumination, with UV light leading to higher yield (0.70 eq CO). This is because the Ru(II) center acts as a trap for the released CO and UV light enables photodissociation of the metal carbonyl moiety. The quantum yield for CO release from [Ru(η^6^-*p*-cymene)Cl(3-Hfl)] upon illumination at 419 nm is 0.001(1) which is among the lowest values measured to date for divalent metal 3-hydroxyflavonolato complexes (Table 1). 

**Scheme 18:**
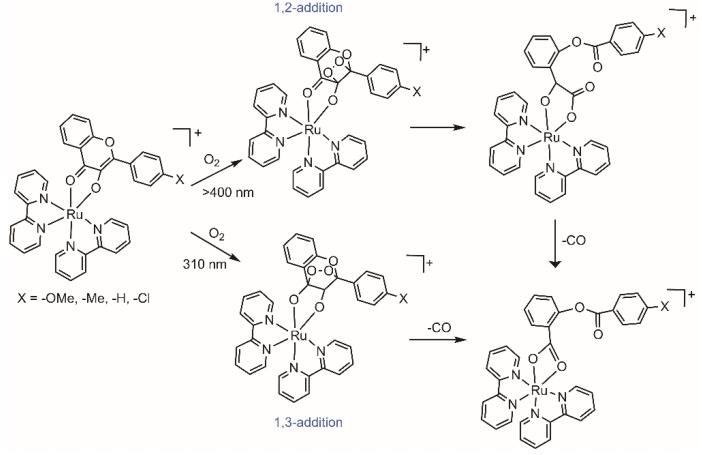
Photoreactivity of Ru^II^ 3-hydroxyflavonolato complexes with O_2_ [109,110].

Farmer, et al. have recently synthesized and characterized a series of Ru(II) bipyridine-ligated 3-hydroxyflavonolato complexes and investigated the mechanistic details of their light-driven reactivity [109,110]. Illumination into distinct excitation bands at low temperature leads to products of two different reaction pathways (Scheme 18). Specifically, upon excitation at wavelengths longer than 400 nm, the complexes undergo 1,2-addition of dioxygen, generating a 1,2-dioxetane intermediate that results in the formation of a Ru^II^-coordinated α-ketoacid complex. This complex is proposed to release CO gas to form a Ru(II) depside complex as determined by ESI-MS and deoxymyoglobin assay. Illumination of the same complex through a 310 nm notch pass optical filter, yields product mixtures derived from 1,3-addition of dioxygen, specifically depside coordinated to Ru(II) and CO. The authors suggest that the observed reactivity patterns are attributed to flavonol tautomeric biradicals [110]. 

## 6. Visible Light-Induced CO Release from Extended 3-Hydroxyflavone and 3-Hydroxybenzo[*g*]quinolone Frameworks

**Scheme 19:**
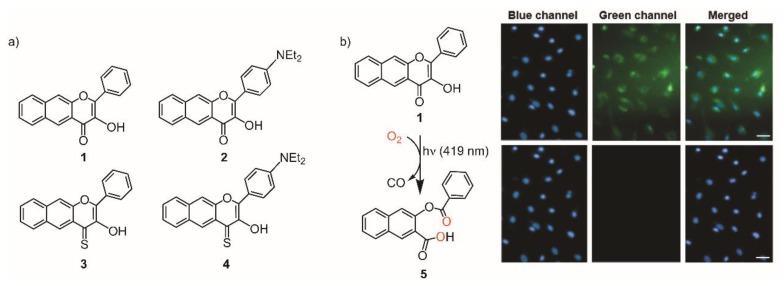
(**a**) Extended 3-hydroxyflavone derivatives (**1**–**4**). (**b**, left) Visible light-induced CO release from **1**. (**b**, right) Individual fluorescence microscopy images of HUVECs incubated with **1** for 4 h. Row 1: Cells exposed to **1** for 4 h. Row 2: Cells from first row illuminated (488 nm light, with a light density of 42,620 lx). Cells were also co-stained with Hoechst 33342 nuclear dye (blue) to assess cell integrity. Size of bar = 50 μm.

We have prepared a series of new flavonols (**1**-**4**) with an additional fused aromatic ring (Scheme 19) to shift the absorption maximum further into the visible range. [111]. Compound **1**, which ionizes partially in DMSO:PBS buffer at pH = 7.4 [112], has been extensively investigated in terms of its O_2_-dependent CO release reactivity. In a range of solvents (Table 2), **1** undergoes dioxygenase-type quantitative visible light-induced (λ_ill_ = 419 or 488 nm) CO release, with the reaction quantum yield (0.006–0.010) depending on the solvent [111,112]. The product generated is an *O*-benzoylsalicylate derivative (**5**). Notably, compound **1** has been recently shown to be amenable to two-photon excitation at 800 nm to trigger CO release [113]. Prior to CO release, compound **1** can be visualized in cells via its green emission at ~580 nm (Scheme 19b). This emission, which is from a tautomeric species resulting from excited state intramolecular proton transfer (ESIPT), is lost upon release of CO, thus enabling tracking of the intracellular CO release process [111,114]. Compound **1** binds weakly to bovine serum albumin (*K*_a_ = 3.2 × 10^3^ M^−1^) at the warfarin binding Site I [112]. This interaction with a protein reduces the quantum yield for CO release (Table 2). Compound **1** is minimally toxic in A549 (IC_50_ = 41 μM), HUVECs (IC_50_ = 82 μM), and RAW 264.7 cells (non toxic up to 100 μM) [111,115,116]. Functionalized derivatives of **1** have been used to probe mitochondrial localization of CO release [115], thiol and H_2_S sensing [117,118] and H_2_O_2_ sensing [113] prior to light-triggered CO release. 

The diethylamino-substituted **2** (Scheme 19a; Table 2) exhibits similar quantitative, O_2_-dependent visible light-induced (λ_ill_ = 419 nm) CO release to that found for **1**. Notably, the sulfur-substituted **3** (Scheme 19a; Table 2) exhibits a much higher quantum yield (0.426(3)) for CO release in CH_3_CN with λ_ill_ > 546 nm under O_2_ [111]. Compound **4** exhibits quantitative O_2_-dependent visible light-induced CO release under aerobic conditions (λ_ill_ > 546 nm) but also produces 0.32(7) equivalents of CO under anaerobic conditions. Both reactions of **4** lead to a mixture of products, with the anaerobic CO release reaction appearing to involve photoisomerization akin to that observed for 3-hydroxyflavone (Scheme 15) [92,93,94]. The mechanism for visible-light induced CO release in **1**–**4** has not been computationally examined but may involve the reactivity of a triplet phototautomer formed via ESIPT reacting with ^3^O_2_ [90,114].

**Scheme 20:**
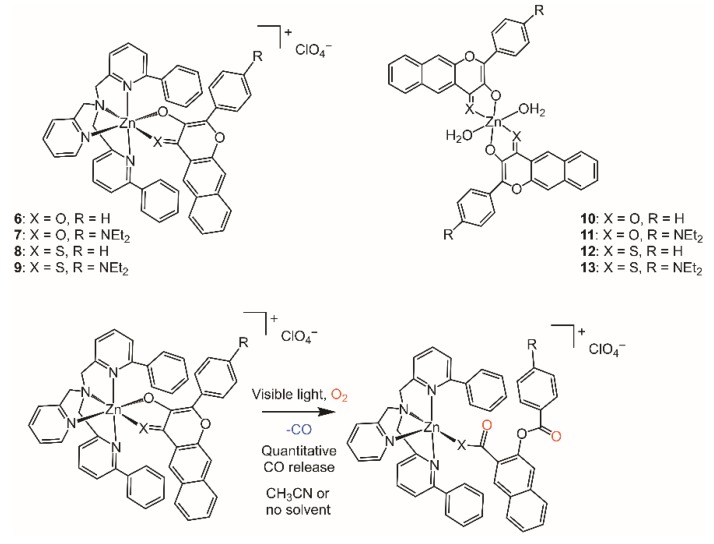
Visible light-induced CO release reactivity of Zn(II) complexes of extended 3-hydroxyflavones [119].

Zinc complexation of **1**–**4** provided the first examples of divalent metal flavonolato complexes (**6**–**13**, Scheme 20) that exhibit O_2_-dependent, quantitative visible light-induced *solid-state* CO release [119]. Similar to the solution reactivity of **1**–**3**, these zinc complexes undergo reaction either in solution or in the solid state to produce Zn(II) *O*-benzoylsalicylate complexes (Scheme 20) and one equivalent of CO per flavonolato ligand. The quantum yields for the reactions of **6**–**9** are enhanced relative to the free flavonols (Table 2). Limited solubility of the Zn(II) bis-flavonolato complexes **10**–**13** in common organic solvents enabled the use of **10** as a visible light driven in situ CO release compound for palladium-catalyzed carbonylation [119]. 

**Scheme 21:**
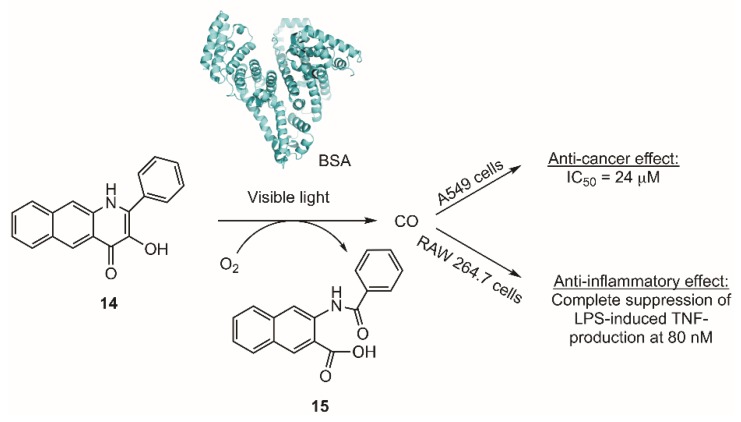
CO release reactivity of **14** in the presence of bovine serum albumin (BSA) produces potent anti-cancer and anti-inflammatory effects [116].

The 3-hydroxybenzo[*g*]quinolone **14** (Scheme 21) was previously reported as a dye [120]. We recently demonstrated that this compound will release one equivalent of CO upon illumination with visible light to produce a non-emissive depside product [116]. Similar to **1**, the presence of **14** in cells can be tracked via fluorescence prior to CO release. Notably, **14** has a high affinity (K_SV_ 2.9 × 10^6^ M^−1^ (SV = Stern-Volmer)) for bovine serum albumin (BSA) protein, binding at Site I (warfarin binding site). The binding affinity of **14** for BSA is 900-fold greater than that of **1**, indicating that small structural changes will dramatically influence protein interactions in these bioinspired compounds. Using the non-covalent protein:**14** adduct for visible light-induced CO delivery to cells, we identified a low IC_50_ value (24 μM) for the eradication of cancer cells (A549) and potent anti-inflammatory effects, as measured by complete suppression of LPS-induced TNF-α production at 80 nM in RAW 264.7 murine macrophage cells [116]. The low IC_50_ value may be due in part to the known effect of serum albumin proteins in facilitating the delivery of small molecules to cancer cells via the enhanced permeability and retention effects [121]. Overall, these initial results indicate that the 3-hydroxybenzo[*g*]quinolone framework has significant potential as a novel CO-releasing motif for applications in biological systems. 

## 7. Concluding Remarks and Future Directions

In this review we have outlined the various reported reaction pathways whereby 3-hydroxyflavone and 3-hydroxy-4-oxoquinoline compounds undergo CO release. Currently there is a strong interest in the development of carbon monoxide-releasing molecules (CORMs) that are metal free [20,122,123], especially those that can be triggered by visible light, which enables localized CO delivery. An additional desired characteristic for CORMs is a chemical framework that can be structurally modified to tune various factors including solubility, the rate of CO release, toxicity, and subcellular or tissue targeting [124]. The studies outlined herein demonstrate that the 3-hydroxyflavone motif will undergo CO release under a variety of reaction conditions, whether metal-coordinated or metal-free, and by either thermal or light-induced reaction pathways. The 3-hydroxyflavone framework is amenable to a variety of structural modifications to tune or augment chemical properties. In this regard, our lab has recently shown that an extended 3-hydroxyflavone motif can be structurally modified to enable sub-cellular targeting [115] and fluorescence-trackable small molecule sensing properties [117,118] prior to CO release. An additional intriguing feature of using 3-hydroxyflavone derivatives as CORMs is the potential of these compounds to exhibit other advantageous properties such as anti-inflammatory, antioxidant and anti-cancer effects [125,126] in addition to CO release. Future work in our laboratory is focused on identifying and investigating these possible dual activity effects. 

Overall, the development of CORMs based on 3-hydroxyflavone and 3-hydroxy-4-oxoquinoline structural frameworks is in its early stages. Using the knowledge of the studies summarized herein we anticipate further development of CORMs based on these biologically-relevant structures. In this regard, a key next step for our laboratory will be evaluation of the CO release reactivity of a range of 3-hydroxyflavone and 3-hydroxy-4-oxoquinoline derivatives under visible light-triggered conditions. Currently, very little is known regarding the structure/reactivity relationships for light-driven reactions of these molecules. If compounds with a range of absorption properties and quantum yields can be developed, this family of compounds could enable controlled, triggered release for the delivery of specific boluses of CO. 

## Figures and Tables

**Table 1:** Quantum yields of UV- or visible light-induced quantitative CO-releasing reactions of divalent metal 3-hydroxyflavonolato complexes.

Compound	Absorption Max. (nm)	Quantum Yield for CO Release	Reference
[(6-Ph_2_TPA)Zn(3-Hfl)]ClO_4_	420	0.09(1) *^a,c^*	[63,95,97]
[(6-Ph_2_TPA)Zn(3-Hfl)]ClO_4_	420	0.012(2) *^b,c^*; 0.006(1) *^b,d^*	[98]
[(6-Ph_2_TPA)Cd(3-Hfl)]ClO_4_	430	0.28(2) *^a,c^*	[95,97]
[(6-Ph_2_TPA)Hg(3-Hfl)]ClO_4_	415	0.31(2) *^a,c^*	[95,97]
[(6-Ph_2_TPA)Mn(3-Hfl)]ClO_4_	415	0.005 *^a,c^*	[96]
[(6-Ph_2_TPA)Co(3-Hfl)]ClO_4_	430	0.005 *^a,c^*	[96]
[(6-Ph_2_TPA)Cu(3-Hfl)]ClO_4_	428	0.005 *^a,c^*	[96]
[(6-Ph_2_TPA)Ni(3-Hfl)]ClO_4_	415	0.008 *^a,c^*	[96]
[(6-Ph_2_TPA)Pb(3-Hfl)]ClO_4_	406	0.21(6) *^a,c^*	[97]
[(TPA)Zn(3-Hfl)]ClO_4_	415	0.006(1)*^b,c^*	[98]
[(bnpapa)Zn(3-Hfl)]ClO_4_	401	0.00027(1) *^b,c^*	[98]
{[(bpy)Zn(3-Hfl)]_2_}(ClO_4_)_2_	414	0.004(1) *^b,c^*	[99]
[Ru(η^6^-*p*-cymene)Cl(3-Hfl)]	472	0.001(1) *^b,c^*	[105]

*^a^* 300 nm; *^b^* 419 nm; *^c^* In CH_3_CN; *^d^* 1:1 DMSO:H_2_O.

**Table 2:** Quantum yields for photoinduced CO release from extended 3-hydroxyflavones, zinc flavonolato complexes, and 3-hydroxybenzo[*g*]quinolone.

Compound	Absorption Maximum (nm)	Quantum Yield for CO Release	Reference
**1**	409 *^b^*	0.007(3) *^a,c^*	[111,112]
	410 *^c^*	0.006(3) *^a,c^*	[112]
	410 *^d^*	0.010(3) *^a,d^*	[112]
	410 *^e^*	0.0063(1) *^a,e^*	[112]
	410 *^F^*	0.0006(1) *^a,f^*	[112]
**2**	442 *^b^*	0.006(1) *^a,b^*	[111]
**3**	478 *^b^*	0.426(3) *^a,b^*	[111]
**4**		Not determined	[111]
**6**	480 *^b^*	0.651(2) *^a,b^*	[119]
**7**	524 *^b^*	0.583(4) *^b,g^*	[119]
**8**	550 *^b^*	0.951(4) *^b,g^*	[119]
**9**	600 *^b^*	0.947(7) *^b,g^*	[119]
**14**	445 *^b^*	0.0045(1) *^a,b^*	[116]

*^a^* 419 nm; *^b^* CH_3_CN; *^c^* 1:1 DMSO:TRIS; *^d^* 1:1 DMSO:PBS; *^e^* 4% DMSO:PBS + CTAB; *^f^* BSA. (40 eq.) in 3.3% DMSO:TRIS; *^g^* White light with 546 nm cut off filters.
